# p53 upregulates PLCε-IP3-Ca^2+^ pathway and inhibits autophagy through its target gene Rap2B

**DOI:** 10.18632/oncotarget.18112

**Published:** 2017-05-23

**Authors:** Jiehui Di, Juanjuan Tang, Heya Qian, Derek A. Franklin, Chad Deisenroth, Yoko Itahana, Junnian Zheng, Yanping Zhang

**Affiliations:** ^1^ Cancer Institute, Xuzhou Medical University, Xuzhou, Jiangsu, P.R. China; ^2^ Jiangsu Center for the Collaboration and Innovation of Cancer Biotherapy, Cancer Institute, Xuzhou Medical University, Xuzhou, Jiangsu, P.R. China; ^3^ Center of Clinical Oncology and Affiliated Hospital of Xuzhou Medical University, Xuzhou Medical University, Xuzhou, Jiangsu, P.R. China; ^4^ Department of Oncology, The Affiliated Hospital of Xuzhou Medical University, Xuzhou Medical University, Xuzhou, Jiangsu, P.R. China; ^5^ Department of Radiation Oncology and Lineberger Comprehensive Cancer Center, School of Medicine, the University of North Carolina at Chapel Hill, Chapel Hill, NC, USA; ^6^ The Hamner Institutes for Health Sciences, Institute for Chemical Safety Sciences, Research Triangle Park, NC, USA; ^7^ Cancer & Stem Cell Biology Program, Duke-NUS Graduate Medical School, Singapore

**Keywords:** p53, Rap2B, PLCε-IP3-Ca^2+^ pathway, autophagy

## Abstract

The tumor suppressor p53 plays a pivotal role in numerous cellular responses as it regulates cell proliferation, metabolism, cellular growth, and autophagy. In order to identify novel p53 target genes, we utilized an unbiased microarray approach and identified *Rap2B* as a robust candidate, which belongs to the Ras-related GTP-binding protein superfamily and exhibits increased expression in various human cancers. We demonstrated that p53 increases the intracellular IP3 and Ca^2+^ levels and decreases the LC3 protein levels through its target gene Rap2B, suggesting that p53 can inhibit the autophagic response triggered by starvation via upregulation of the Rap2B-PLCε-IP3-Ca^2+^ pathway. As a confirmed target gene of p53, we believe that further investigating potential functions of Rap2B in autophagy and tumorigenesis will provide a novel strategy for cancer therapy.

## INTRODUCTION

p53 is regarded as a central stress sensor and a DNA sequence-specific transcription factor [1]. p53 is stabilized in response to various stresses including oxidative stress, genotoxic damage, and oncogenic growth signals before regulating numerous downstream target genes that exert various cellular responses dependent on both the type of stress and cellular context [2-4]. Previous studies have established the vital role of p53 in regulation of cell cycle arrest, senescence, DNA repair, apoptosis, and autophagy [5-7]. However, the exact mechanisms as to how p53 exerts so many diverse effects remain unclear. The current understanding of p53-mediated autophagic regulation is particularly complex and ambiguous. Therefore, even though a significant list of p53 target genes has been detailed previously [8], it is still of great importance to identify and study novel p53 downstream targets in order to gain a more complete understanding of the p53 stress response pathway. Here, we confirm Rap2B as a target gene of p53 that is involved in the regulation of p53 induced autophagy.

Rap2B, a Ras family protein, was initially discovered from a screened platelet cDNA library in the early 1990s [9]. We found that Rap2B was highly expressed in multiple human cancers. Previous studies have determined that in response to activation of the EGF receptor Rap2B promotes the activation of phospholipase C-ε (PLCε), a pivotal enzyme localized to the plasma membrane, eliciting a range of biological functions [10-13]. In previous studies, we showed that Rap2B could be induced by nocodazole treatment to regulate the cytoskeleton in a p53-dependent fashion. Additionally, we demonstrated that Rap2B promoted migration and invasion of human breast cancer and suprarenal epithelioma [7, 14].Significantly, Mestre et al. have reported that Rap2b along with cAMP, EPAC, and calpain activation negatively regulate Hla-induced autophagy, indicating that Rap2B is involved in the autophagic pathway [15].

Cell stresses, such as nutrient deprivation, oxidative stress, and the accumulation of damaged organelles, activate autophagy to mediate cellular self-catabolism and recycling through lysosomes, thus maintaining homeostasis as well as survival [16, 17]. Genetic analysis has resulted in the discovery of several autophagy-related genes (ATGs), which play key roles in facilitating autophagosome formation and the regulation of autophagy [16, 18]. The mechanisms by which autophagy is regulated are complicated with inputs from various signaling cascades including p53 and some of its transcriptional target genes [2, 7, 19]. Nuclear p53 enhances autophagy primarily through the activation of AMP-kinase (AMPK) resulting in the downregulation of mammalian target of rapamycin (mTOR), which functions as an inhibitor of autophagy. Conversely, cytoplasmic p53 can inhibit autophagy [20]. In previous publications [21-24], it was revealed that reducing intracellular inositol triphosphate (IP3) levels or genetic knockdown of the IP3 receptor induces autophagy independent of mTOR regulation via Bcl-2-mediated release of Beclin 1, thus linking IP3 with the regulation of autophagy. Furthermore, Mestre et al. suggest that Rap2B links both the classical autophagic pathway induced by starvation and the ‘‘non-canonical’’ autophagy pathway triggered by toxins [15].

Based on the research detailed above, we expect that p53, through its target gene Rap2B, participates in the regulation of the autophagic response. In the present study, we demonstrate that Rap2B is an effector of p53 stress response by upregulating the PLCε-IP3-Ca^2+^ pathway and inhibiting autophagy triggered by starvation.

## RESULTS

### Rap2B is a p53 transcriptional target

A previous study by our laboratory has demonstrated that the expression of p53 can be controlled by manipulating Mdm2 expression through the generation of mice bearing Mdm2 WT (+/+), null (-/-), or RING finger C462A point mutation (m/m) alleles [25]. In addition, the switchable *p53ER*^*TAM*^ (p53ER hereafter) allele was introduced into mice to facilitate modulation of p53 transactivation function in which the endogenous *Trp53* (p53) gene is replaced by one encoding *p53ER*. p53ER expresses a full-length p53 protein fused C-terminally with the hormone binding domain of a modified estrogen receptor and is transcriptionally regulated in the same manner as WT p53. Therefore, p53ER activity can be turned ‘‘on’’ or ‘‘off’’ by administration or withdrawal of 4-hydroxytamoxifen (4-OHT) in this inducible system [26].

**Figure 1:**
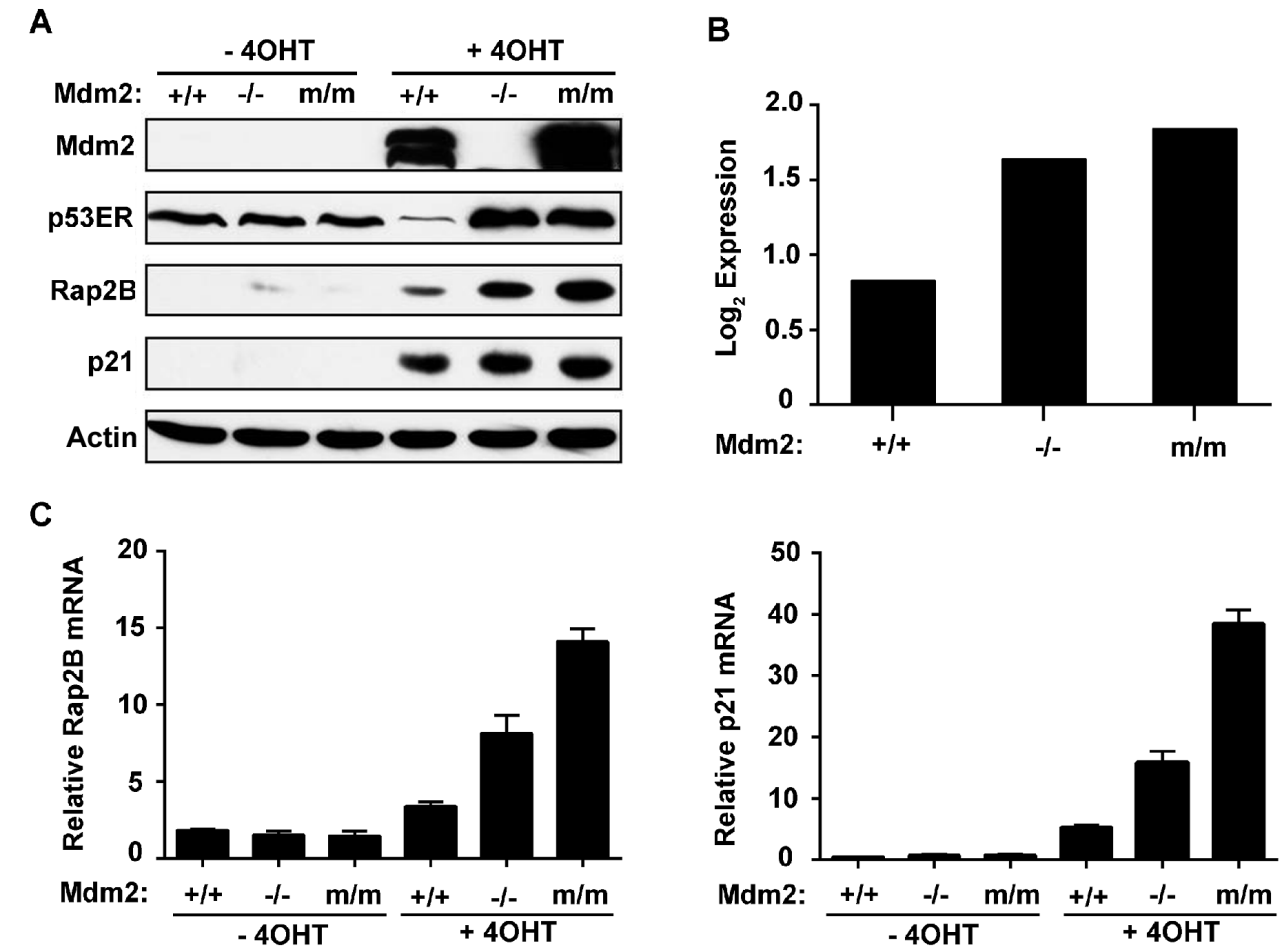
Rap2B is a p53 transcriptional target **A.** Mouse embryonic fibroblasts harboring a single p53ER fusion allele and a p53 null allele (p53ER/- MEF) cell lines with the indicated Mdm2 genotypes including Mdm2 +/+, Mdm2 -/-, or C462A (m/m) Mdm2 were treated for 24 h with 4-OHT. Cells were harvested and analyzed by western blot. **B.** Corresponding microarray Log2 expression values of p53ER/- MEF cell lines following 24 h treatment with 4-OHT. **C.** p53ER/- MEF cell lines with the indicated Mdm2 genotypes were treated for 24 h with 4-OHT. Cells were harvested and analyzed by real-time PCR for expression of *Rap2B* and *p21* mRNA.

We previously observed the transactivation ability of p53ER to be low, medium, and high across Mdm2 WT, null, and C462A (m/m) MEF cells respectively, which provides a unique system to study p53 transactivation potential at close to physiological levels. We then performed a microarray experiment screening for novel p53 candidate target genes by using these *p53ER* MEF cell lines [25]. We identified a large number of the genes identified previously in a ChIP-chip array [27]. Rationally, candidate genes should meet the arbitrary low-medium-high Log_2_ expression threshold. One such candidate gene was Rap2B, a member of the Ras family; moreover, we found that most carcinoma cells expressed higher levels of Rap2B than in the non-cancerous immortalized BJ or WI-38 cell lines (Supplementary Figure 1). Although Rap2B induction was low in the Mdm2 WT MEF cells after p53 activation, the Mdm2 null and C462A (m/m) MEF cells exhibited comparatively moderate-to-high expression levels of Rap2B (Figure 1A). Rap2B protein levels induced by 4-OHT mirrored the Log2 expression values indicated by the microarray (Figure 1B), thereby providing a strong correlation between transcript and protein levels. To confirm the result from the microarray, we performed real-time PCR to monitor the induction of Rap2B mRNA in the above MEF cells treated with or without 4-OHT. Our results showed that the induction of Rap2B mRNA was p53-dependent with the canonical p53 target gene *Cdkn1a* (p21) as a positive control (Figure 1C).

### Rap2B gene locus contains p53-binding sites

**Figure 2:**
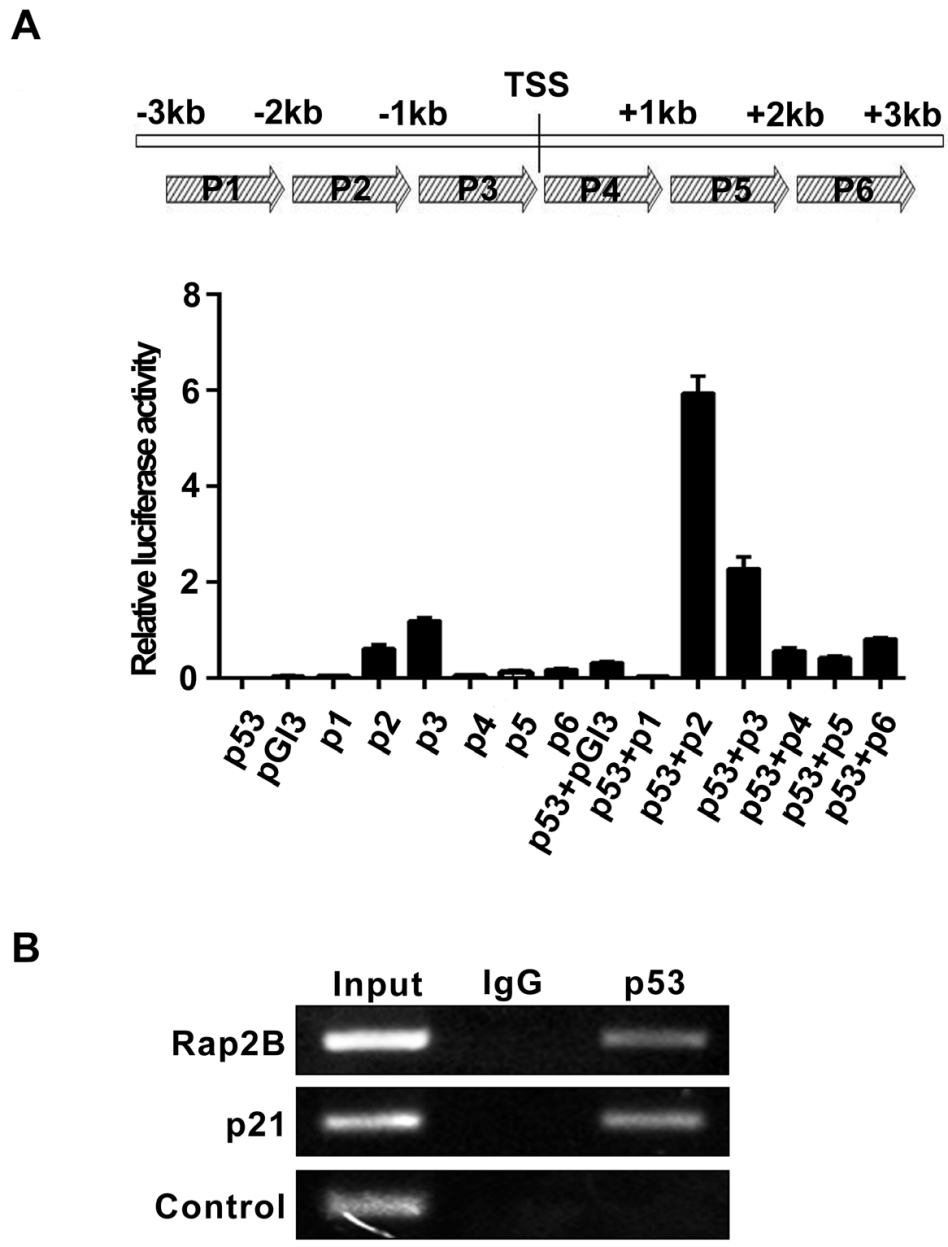
Rap2B gene locus contains p53-binding sites **A.** Luciferase reporter assay to measure the induction of Rap2B promoter by p53. **B.** Chromatin immunoprecipitation (ChIP) assay to measure the binding of p53 in the promoter region of the *Rap2B* gene in p53ER/- MEF cells treated with 4-OHT.

To determine whether Rap2B is a direct target of p53, we carried out a heterologous promoter-reporter assay using a luciferase vector pGl3-Rap2B-p(1-6) (p1-6 corresponding to -3,000∼+3,000 bp to the transcriptional start site), which was prepared by cloning the nucleotide sequence around the Rap2B promoter. Figure 2A shows a p53 dependent increase in luciferase activity from pGl3-Rap2B-p2 when compared to the empty vector alone. We then searched for a consensus p53-binding sequence within the genomic locus containing the human *Rap2B* gene. A single potential binding site (designated p2, corresponding to -2,000 ∼-1,000 bp to the TSS) was identified consisting of two copies of the 10-bp consensus p53-binding motif and the sequence is well conserved between mouse and human (Supplementary Figure 2).

We then utilized an *in vivo* chromatin immunoprecipitation assay (ChIP) to confirm the direct binding of p53 on the *Rap2b* gene. ChIP assays using anti-p53 antibodies revealed that a DNA fragment containing the p2 sequence was reproducibly present in the immunoprecipitated complex containing the p53 protein, indicating that p53 binds to the p2 site *in vivo*. As a positive control for the ChIP assay, we found that p53 strongly binds to the p21 promoter (Figure 2B). Thus, our findings provide clear evidence that there is a functional p53 response element in the promoter region of the *Rap2B* gene.

### ActD and UV induce Rap2B in WT, but not p53-null, MEF or HCT116 cells

**Figure 3:**
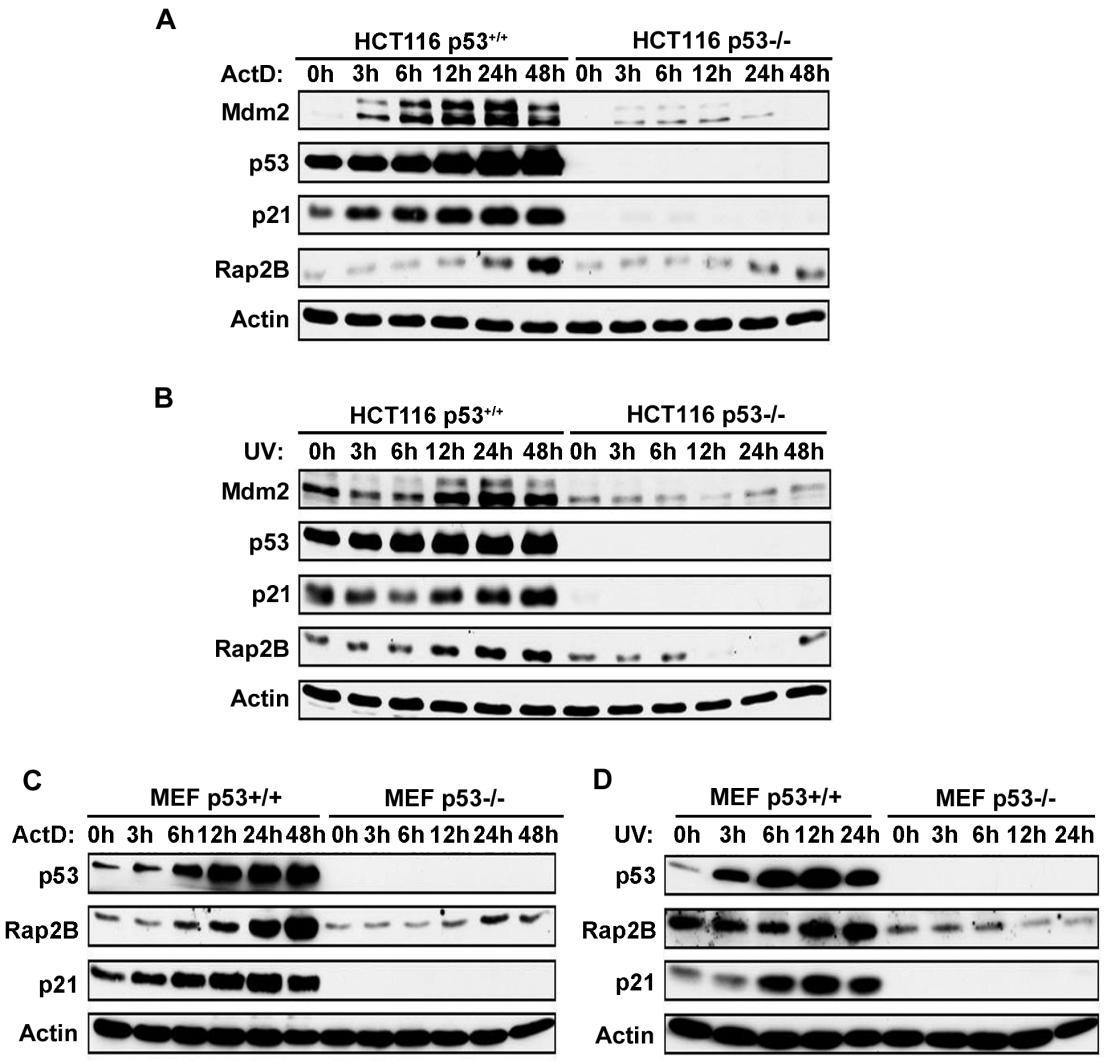
ActD and UV induce Rap2B in WT, but not p53-null, MEF or HCT116 cells **A.**, **B.** WT and p53-null HCT116 cells were treated with 5 nM ActD or 40J UV for various times. Cells were harvested and analyzed by western blot. **C.**, **D.** WT and p53-null MEF cells were treated with 5 nM ActD or 40J UV for various times followed by western blot.

Since p53 is established as a stress sensor that is activated by diverse stimuli [4], we investigated whether Rap2B could be induced in response to diverse stresses in a p53 dependent manner. Actinomycin D (ActD) has been used as a chemotherapeutic drug in the treatment of a variety of human cancers [28]. At high concentrations (e.g. >30 nM) of ActD causes DNA damage and inhibits transcription from all three classes of RNA polymerases, whereas at low concentrations (e.g. <10 nM) ActD does not cause DNA damage but selectively inhibits RNA pol I-dependent transcription to directly shut down ribosomal biogenesis [29, 30]. Therefore, in addition to DNA damage in response to UV treatment, we also use non-genotoxic doses of ActD (5 nM) to activate p53. MEF (*p53*^*+/+*^, *p53*^*−/−*^) and HCT116 (*p53*^*+/+*^, *p53*^*−/−*^) cells were treated with/without ActD or UV followed by western blot analysis to evaluate Rap2B and p53 protein levels. As shown in Figure 3, both ActD and UV promoted the stabilization and activation of p53 in both MEF and HCT116 *p53*^*+/+*^ cells as expected. Accordingly, the protein levels of Rap2B increased significantly in *p53*^*+/+*^ cells treated with ActD or UV, but not in *p53*^*-/-*^ cells. Our results indicate that Rap2B is a p53 inducible target and can be activated by ribosomal biogenesis stress and DNA damage. However, it is important to note that the protein levels of Rap2B increased slightly at 24 h in p53^-/-^ cells treated with ActD, which suggests that a portion of the Rap2B protein expression in response to ribosomal stress could be p53 independent.

### p53 increases the intracellular IP3 level through Rap2B

**Figure 4:**
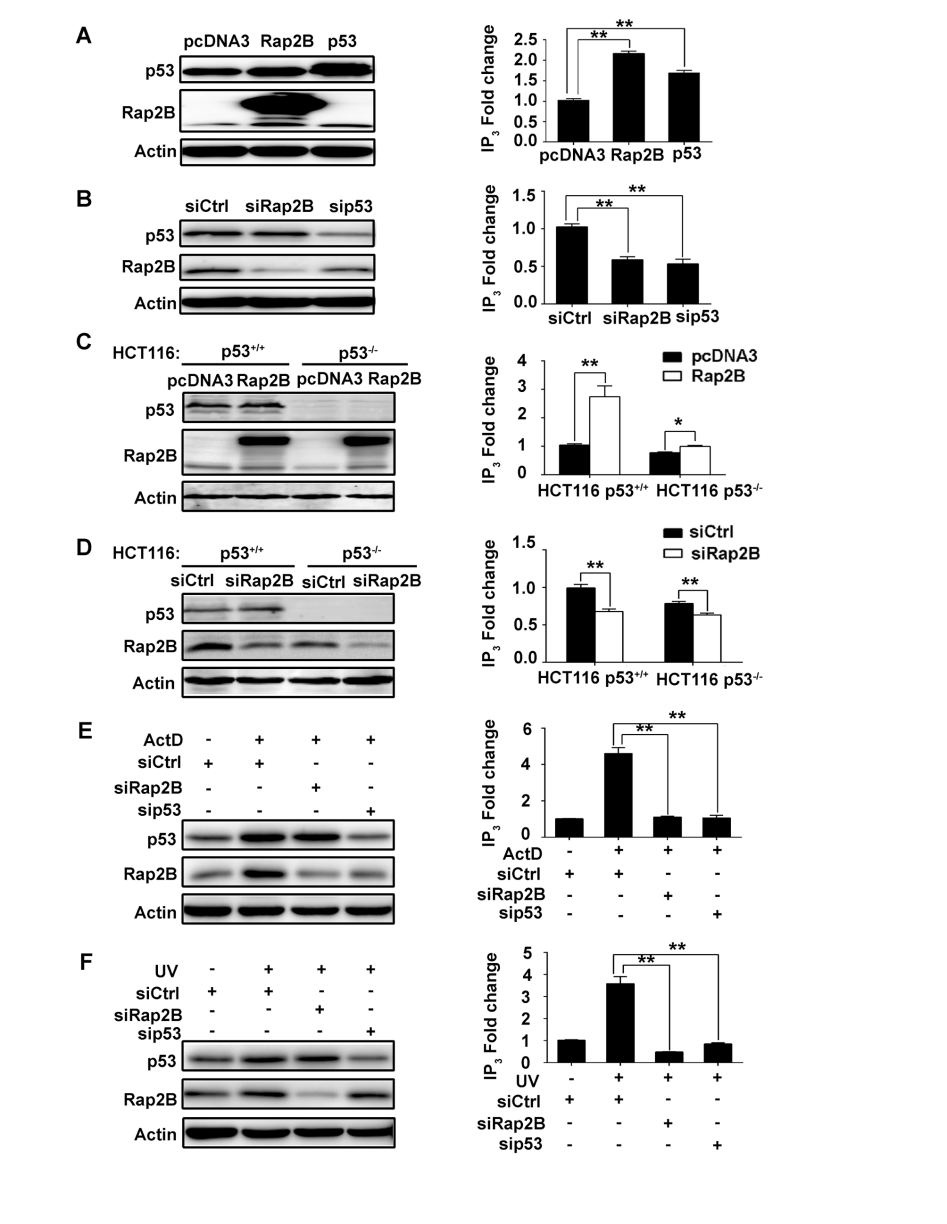
p53 can increase the intracellular IP3 level through Rap2B in U2OS and HCT116 cells **A.**, **C.** U2OS cells or HCT116 (p53^+/+^, p53^-/-^) cells were transfected with myc tagged Rap2B, p53 and control plasmids. The protein levels of Rap2B and p53 and the intracellular IP3 levels were measured by western blot analysis and an IP3 ELISA Kit, respectively. **B.**, **D.** U2OS cells or HCT116 (p53^+/+^, p53^-/-^) cells were transfected with siRNA against *Rap2B* and *p53*, the protein levels of Rap2B and p53 and the intracelluar IP3 levels were measured by western blot analysis and an IP3 ELISA Kit, respectively. **E.**, **F.** U2OS cells were transfected with siRNA against *Rap2B* and *p53*, cells were then either treated or untreated with 5 nM ActD or 40J UV for 4 h, the protein levels of Rap2B and p53 and the intracelluar IP3 levels were measured by western blot analysis and an IP3 ELISA Kit. All experiments were carried out in triplicate. Data are presented as mean ± SD (*n* = 3). *P < 0.05, **P< 0.01 in comparison with respective control group.

Previous studies [11, 31] have established that Rap2B induces PLCε stimulation. Stimulation of PLCε catalyzes the breakdown of phosphatidylinositol 4,5-bisphosphate (PIP2) into the second messengers diacylglycerol (DAG) and inositol 1,4,5-trisphosphate (IP3), which activate protein kinase C and Ca^2+^ release from intracellular stores, leading to a cascade of intracellular events including regulation of cell growth, cell differentiation, and gene expression [32, 33]. Therefore, we performed ELISA to determine the intracellular IP_3_ levels in U2OS and HCT116 cells under various levels of Rap2B and p53. The knockdown or overexpression of Rap2B and p53 was determined by western blot as shown in Figure 4, left panels. Our results revealed that intracellular IP3 levels were increased after exogenous expression of p53 or Rap2B in U2OS cells (Figure 4A, right panels), whereas knockdown of either p53 or Rap2B decreased intracellular IP3 levels (Figure 4B, right panels). This direct relationship between Rap2B expression and IP3 levels was also observed in HCT116 cells (Figure 4C, 4D, right panel). Additionally, we detected decreased levels of IP3 in HCT116 *p53*^*-/-*^ cells compared with *p53*^*+/+*^ cells (Figure 4C, 4D, right panel). Furthermore, we utilized ActD or UV treatment to confirm whether p53 increases the intracellular IP3 level through Rap2B. U2OS cells were transfected with siRNA against *Rap2B* and *p53*, and then treated with/without ActD or UV. The protein levels of Rap2B and p53 were detected by western blot analysis. In response to both ActD and UV driven p53 activation, the expression level of Rap2B was increased, while knocking down p53 decreased the observed levels of Rap2B significantly (Figure 4E, 4F, left panels). Accordingly, the level of IP3 was also distinctly increased when treated with ActD or UV and abrogated by knocking down either Rap2B or p53 in both cell lines (Figure 4E, 4F, right panels). Similar results were also observed in HCT116 cells (Supplementary Figure 3A, 3B). These results indicate that p53 increases the intracellular IP3 level through a Rap2B-dependent mechanism.

### p53 increases the intracellular Ca^2+^ level through Rap2B

**Figure 5:**
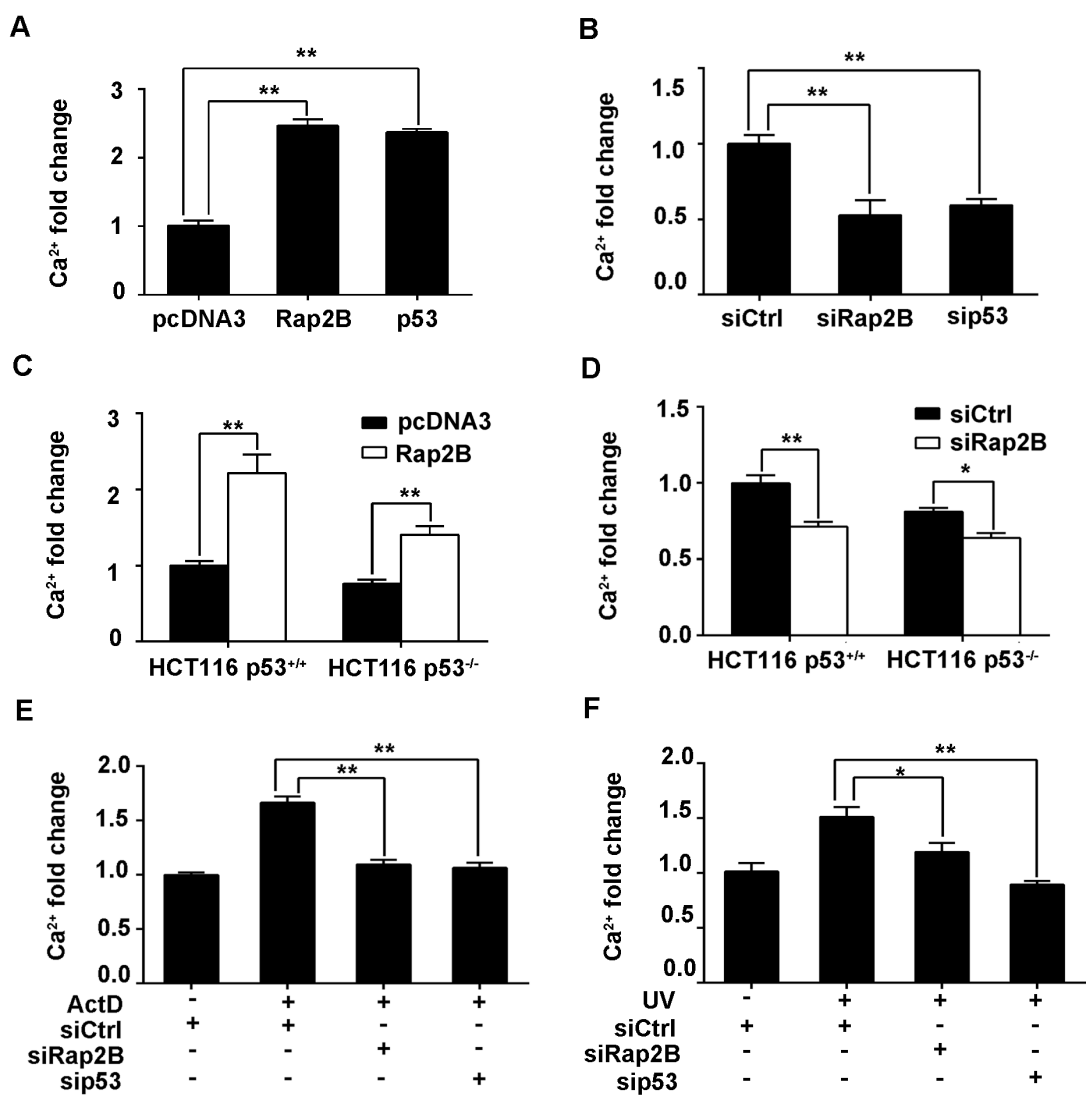
p53 can increase the intracellular Ca^2+^ level through Rap2B in U2OS and HCT116 cells **A.**, **C.** After U2OS cells or HCT116 (p53^+/+^, p53^-/-^) cells were transfected with Rap2B plasmids, p53 plasmids and control plasmids, the intracellular Ca^2+^ levels were measured by flow cytometry. **B.**, **D.** After U2OS or HCT116 (p53^+/+^, p53^-/-^) cells were transfected with Rap2B siRNA, p53 siRNA and control siRNA, the intracellular Ca^2+^ levels were measured by flow cytometry. **E.**, **F.** After U2OS cells were transfected, cells were either treated or untreated with 5 nM ActD or 40J UV for 4 h, the intracellular Ca^2+^ levels were measured by flow cytometry. All experiments were carried out in triplicate. Data are presented as mean ± SD (*n* = 3). *P < 0.05, **P < 0.01in comparison with respective control group.

IP3 interacts with a specific receptor located on the surface of the endoplasmic reticulum, resulting in a rapid rise in intracellular Ca^2+^ concentrations, which plays a crucial role in numerous cellular responses such as cell migration, apoptosis and autophagy [34, 35]. In order to determine whether the p53-Rap2B pathway regulates intracellular Ca^2+^ levels, we performed flow cytometry in transfected U2OS and HCT116 cells. As shown in Figure 5A, overexpression of Rap2B or p53 was sufficient to increase the intracellular Ca^2+^ level in U2OS cells. In contrast, decreased levels of Ca^2+^ were detected after knocking down Rap2B or p53 (Figure 5B). Moreover, we observed an increase in Ca^2+^ levels in HCT116 cells transfected with Rap2B plasmid and the expected decrease in Ca^2+^ levels after knockdown of Rap2B. Predictably, the Ca^2+^ levels of *p53*^*-/-*^ cells were lower than that of *p53*^*+/+*^ in HCT116 cells (Figure 5C and 5D). In Figure 4/5D, IP3/Ca^2+^ was also decreased in response to Rap2B knockdown even in HCT116 *p53*^*-/-*^ cells, which suggest that Rap2B could also mediate IP3 induction independent of p53. Additionally, both cell lines treated with ActD or UV displayed the expected increased levels of Ca^2+^, but these effects were blocked by targeting *Rap2B* or *p53* with specific siRNAs (Figure 5E, F and Supplementary Figure 4A, 4B). Taken together, our data show that Rap2B is a central participant in the regulation of p53-mediated increases of intracellular Ca^2+^.

### p53 decreases the LC3 protein levels through Rap2B

The data shown above indicate that p53 increases the intracellular IP3 and Ca^2+^ levels through its transcriptional target Rap2B. Mestre *et al.* [15] demonstrated that Rap2B was involved in the regulation of autophagy, and further proposed a model by which cAMP inhibits *S. aureus*-induced autophagy through the participation of the Epac/Rap2b/PLCε pathway and calpain activation. Specifically, when activated by cAMP/EPAC, Rap2B activates PLCε and induces an increase in IP3, which leads to Ca^2+^ release from the endoplasmic reticulum and then activates the calpain cysteine-proteases. These calpains are able to cleave Atg5 inhibiting the autophagic pathway. Therefore, we wanted to determine whether the p53-Rap2B pathway participates in the regulation of autophagy induced by starvation in U2OS and HCT116 cells by tracking the conversion of LC3-I to LC3-II.

**Figure 6:**
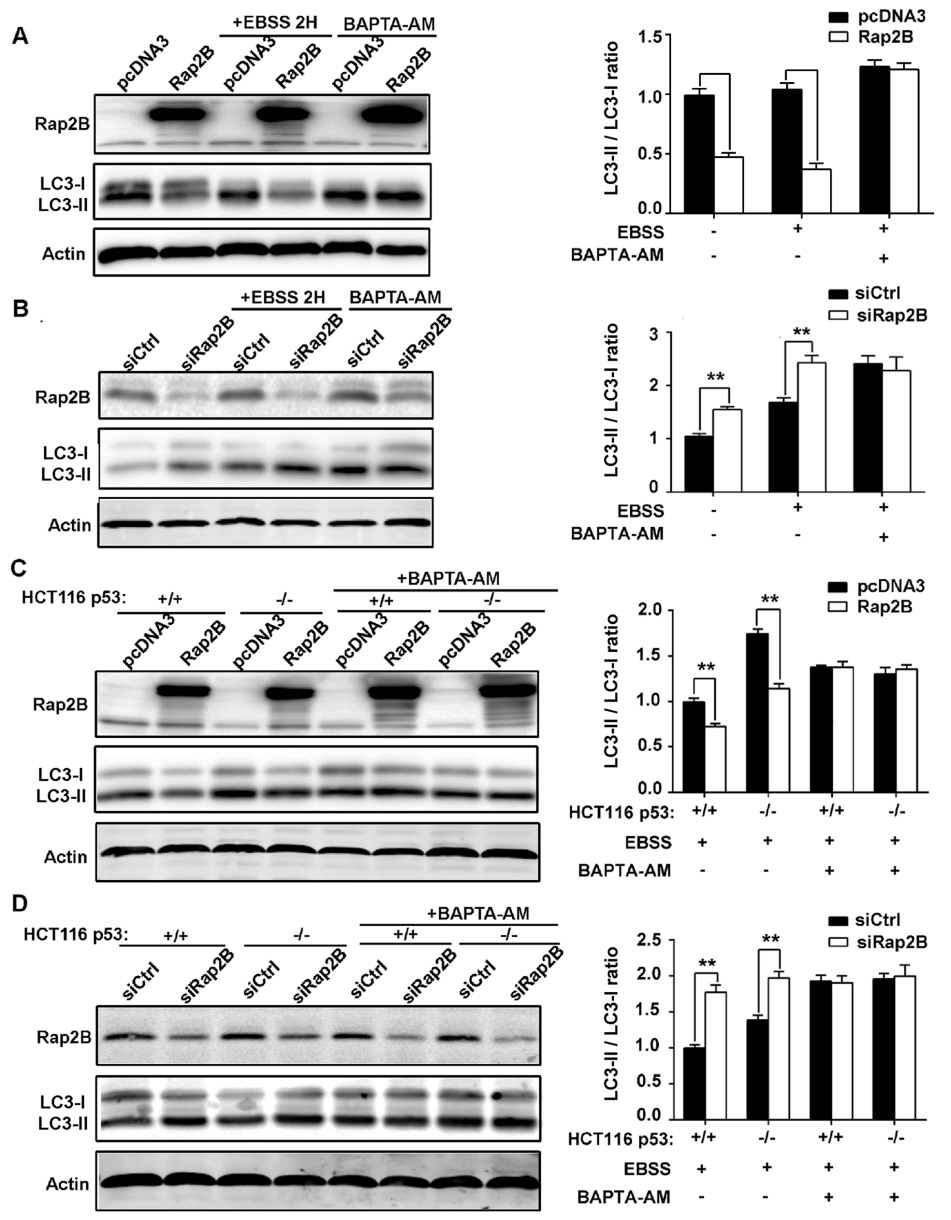
p53 can decrease the LC3 protein level through Rap2B, and BAPTA-AM can disrupt the inhibition **A.** After U2OS cells were transfected with Rap2B plasmids and control plasmids, cells were either treated with EBSS for starvation or untreated. Then, starved cells were incubated in the presence or absence of BAPTA-AM (30μM) for 30 min. The protein levels of Rap2B, LC3, and Actin were measured by Western blot in Rap2B overexpression and control group. **B.** After U2OS cells were transfected with Rap2B siRNA and control siRNA, cells were either treated with EBSS for starvation or untreated. Then, starved cells were incubated in the presence or absence of BAPTA-AM (30μM) for 30 min. The protein levels of Rap2B, LC3, and Actin were measured by Western blot in Rap2B overexpression and control group. **C.**, **D.** After transfection and starvation, HCT116 cells were incubated in the presence or absence of BAPTA-AM (30μM) for 30 min, the protein levels of Rap2B, LC3, and Actin were measured by Western blot. All experiments were carried out in triplicate. Data are presented as mean ± SD (*n* = 3). **P< 0.01 in comparison with respective control group.

Both cell lines were transfected with either Rap2B plasmid or Rap2B siRNA before being subjected to starvation conditions with Earle’s balanced salts solution (EBSS), which contains no nutrients except for glucose, or incubated in complete medium for two hours. Next, we carried out western blot analysis to determine the expression of lipidated LC3 (LC3-II), an autophagic marker. In U2OS cells with pcDNA3 or control siRNA transfection, EBSS treatment increased the conversion of endogenous LC3-I to LC3-II. However, the EBSS-induced autophagic effect was substantially minimized by Rap2B overexpression, while knocking down Rap2B also dramatically increased LC3-I to LC3-II conversion (Figure 6A, 6B). Similar results were obtained in HCT116 cells, and we also found an increased level of LC3-II in HCT116 p53^−/−^ cells compared with HCT116 p53^+/+^ cells, indicating that deletion of p53 facilitated the activation of autophagy (Figure 6C, 6D).

To further verify that Ca^2+^ participates in p53-mediated autophagy inhibition through Rap2B, we assessed the effect of BAPTA-AM, a Ca^2+^ chelator, on the autophagic response induced by starvation. We incubated the transfected cells in the presence or absence of BAPTA-AM (30μM) for 30 min before exposing the cells to EBSS. Our results indicate that while EBSS alone was able to induce autophagy that can be downregulated by p53 and Rap2B, these effects could be abolished by BAPTA-AM, further confirming the important role Ca^2+^ plays in the autophagic pathway (Figure 6). Collectively, these results indicate that p53 negatively regulates this autophagic response induced by starvation through Rap2B via Ca^2+^ signaling.

## DISCUSSION

In response to multiple cellular stresses, the p53 pathway is activated to achieve various functions through transactivation of numerous target genes [2]. However, the particular molecular mechanisms facilitating these versatile functions are still not clearly defined. Although p53 target genes have been well documented in recent years, further study of both p53 and novel target genes is still necessary. By studying p53 transcription target gene microarray data in this study, we have confirmed Rap2B as a novel p53 target (Figures 1-3). Furthermore, we show an important role for Rap2B in autophagy regulation. Specifically, Rap2B is able to upregulate the PLCε-IP3-Ca^2+^ pathway and inhibit autophagy induced by starvation. Rap2B is a Ras family protein and is upregulated in many tumors, which could be a new therapeutic target for cancer.

Besides the canonical function of p53 as a tumor suppressor, an increasing number of new roles for p53 have recently been established including the ability to regulate autophagy [2, 19]. During the past few years, it has been reported that p53 plays a complex role in the regulation of autophagy that depends on its subcellular localization. Nuclear p53 predominantly induces autophagy by transcriptional regulation of several pro-autophagic factors that activate AMPK, a positive regulator of autophagy, while simultaneously down-regulating the negative regulator of autophagy, mTOR [36, 37]. However, cytoplasmic p53 largely inhibits autophagy. p53-induced glycolysis and apoptosis regulator (TIGAR) [38] suppresses autophagy by inhibiting ROS in response to metabolic stress or nutrient deprivation [2]. These data indicate that many target genes of p53 are involved in the regulation of autophagy.

In this study, we confirmed Rap2B as a target gene of p53 and demonstrated that p53-induced Rap2B could negatively regulate autophagy triggered by starvation. Our data also suggest that p53-Rap2B increases the levels of intracellular IP3 and Ca^2+^ levels (Figure 4, 5). Consistent with our results, Mestre et al. have shown that the direct activation of Rap2B by cAMP was sufficient to inhibit autophagy induced by α-Hemolysin independent of the mTOR pathway, but dependent on the autophagic protein Atg5 [15]. Additionally, Rubinsztein and coworkers [39] demonstrated the importance of EPAC and its effector Rap2B in the regulation of autophagy. This study elucidated an mTOR-independent pathway regulating autophagy in which the direct activation of EPAC/Rap2B by cAMP activates PLCε to upregulate IP3 levels, which induces the release of Ca^2+^ from the endoplasmic reticulum. Multiple report shave shown that Rap2B stimulates PLCε and induces Ca^2+^ increase [31, 40, 41], which activates the calcium-dependent cysteine-protease calpains [42]. The activated calpains in turn are able to cleave Atg5 inhibiting the autophagic pathway. We speculate that the PLCε-IP3-Ca^2+^ pathway might be closely associated with p53 dependent inhibition of autophagy via Rap2B which is connected to the key autophagic protein Atg5, although the precise mechanism remains to be elucidated.

To our knowledge, this study is the first to demonstrate that p53-Rap2B signaling pathway plays an effectively inhibitory effect on the starvation-induced autophagic response by upregulating PLCε-IP3-Ca^2+^ pathway. In support of this finding, a previous report has shown that reintroduction of wild-type p53 to HCT116 *p53*^*−/−*^ cells decreases baseline levels of autophagy [43]. Consistently, in our experiments, elevated levels of LC3-II were observed in HCT116 *p53*^*−/−*^ cells relative to *p53*^*+/+*^ cells, indicating that p53 can be an efficient inhibitor of autophagy. Meanwhile, we also observed that the inhibition of Ca^2+^ signaling by the Ca^2+^ chelating agent BAPTA-AM abolished the inhibitory effect of p53-Rap2B signaling on autophagy induced by starvation, which is similar to previous findings. Thus, it is likely that the intracellular Ca^2+^ levels are involved in the regulation of autophagy mediated by p53-Rap2B pathway.

**Figure 7:**
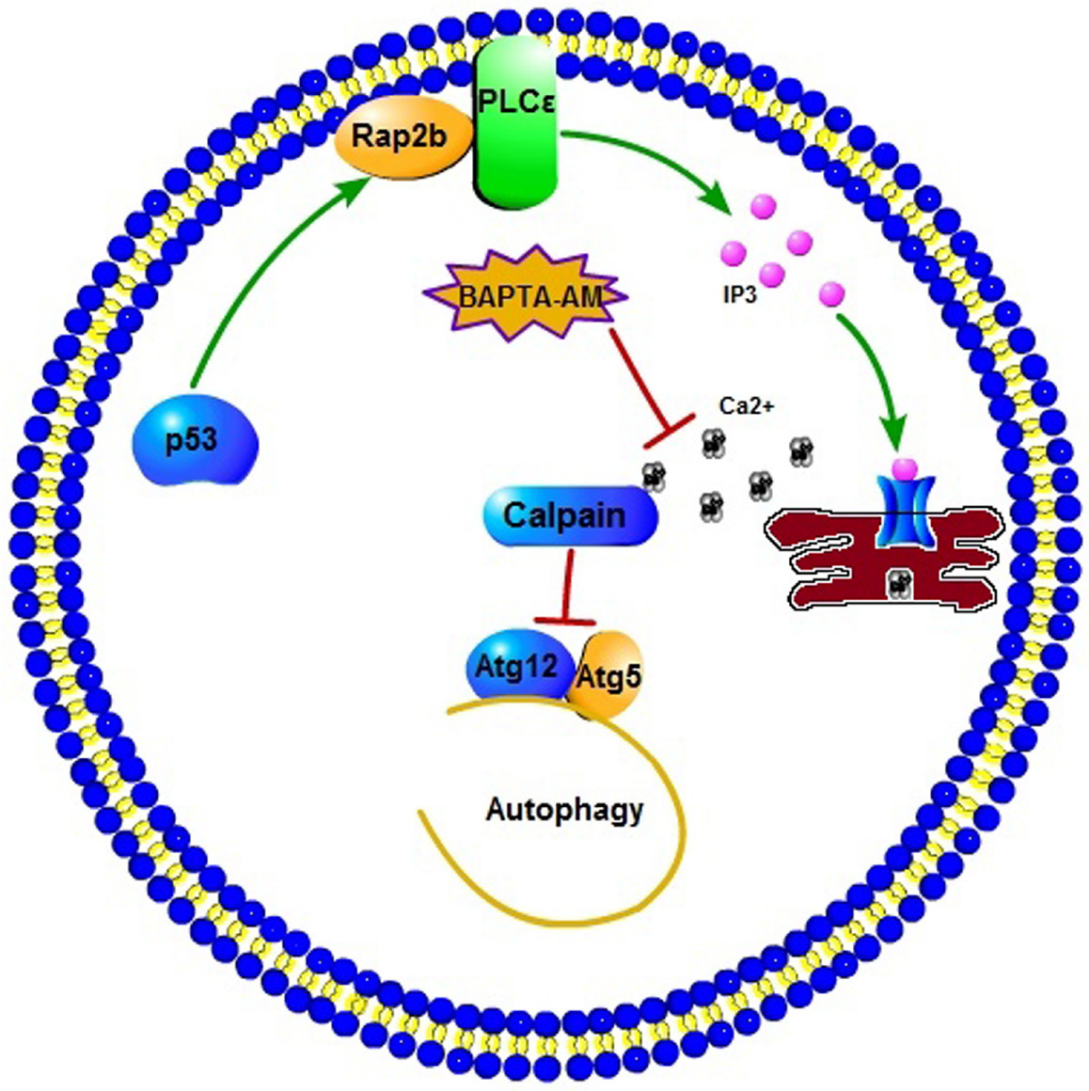
A schematic diagram illustrating the proposed p53-induced Rap2B upregulates PLCε-IP3-Ca^2+^ pathway and inhibits autophagy p53-induced Rap2B can increase the intracellular IP3 and calcium level. The elevated calcium subsequently activates calpains, which are able to cleave Atg5, inhibiting autophagy by decreasing the levels of Atg12-Atg5 conjugate. The calcium chelator BAPTA-AM could block this effect.

In summary, our results showed that Rap2B is a direct p53 target gene, through which p53 upregulates the PLCε-IP3-Ca^2+^ pathway to inhibit autophagy. Moreover, we speculate that the influence of p53-Rap2B signal on autophagy is connected to the key autophagic protein Atg5, rather than via the classical autophagy machinery based on the AMPK-dependent inhibition of mTOR, as shown in Figure 7. Given the established role of p53 in autophagy regulation and tumor suppression, as well as the validation of Rap2B as a p53 target gene, any potential interplay between p53, Rap2B, autophagy and tumor metabolism deserves further investigation. Further experiments are underway to explore the effect of p53-mediated inhibition of autophagy through Rap2B on tumorigenesis, cancer cell proliferation, and migration by regulating the PLCε-IP3-Ca^2+^ signaling pathway using an *in vivo* tumor mouse model. Building on this study and other key findings we expect to develop potentially revolutionary new strategies for cancer therapy.

## MATERIALS AND METHODS

### Cell culture and transfection

U2OS, HCT116 and mouse embryo fibroblasts (MEF) cell lines were maintained in Dulbecco’s modified Eagle’s medium supplemented with 10% fetal bovine serum, L-glutamine, 100μg/ml penicillin, and 100μg/ml streptomycin at 5% CO_2_ in a humidified chamber. Nonspecific control siRNA, p53 or Rap2B siRNA (Invitrogen, Shanghai, China) was transfected into U2OS and HCT116 cells at 60% confluence by siLentFect Lipid Reagent (Bio-Rad, Hercules, CA) according to the manufacturer’s protocol. The pcDNA3-Myc3-control, pcDNA3-p53 and pcDNA3-Myc3-Rap2B plasmids were constructed previously in our lab. Cells were grown to 90% confluence before being transiently transfected with Rap2B plasmids using Lipofectamine 2000 (Invitrogen, Shanghai, China) according to the manufacturer’s protocol.

### Microarray analysis

Total RNA was isolated from MEFs treated with 4-hydroxytamoxifen (4-OHT) for 12 and 24 h using an RNeasy kit (Qiagen). One microgram of total RNA was amplified and labeled using the low RNA input linear amplification kit (Agilent Technologies, Wilmington, DE), and hybridization was performed on Agilent 4×44 K mouse whole genome DNA microarrays. Arrays were washed and scanned using an Agilent scanner (Agilent Technologies). Genes that were significantly up or down-regulated were identified using significance analysis of microarrays.

### Chromatin immunoprecipitation (ChIP) assay

The ChIP assays were performed by using the ChIP Assay Kit (Upstate, Lake Placid, NY) with antibodies against mouse p53 (Santa Cruz, M19, Dallas, TX). The cells (2×10^6^) were cross-linked with a 1% formaldehyde solution for 15 min at 37°. The cells were then lysed in 200 ml of SDS lysis buffer and were sonicated to generate 300-800 bp DNA fragments. After centrifugation, the cleared supernatant was diluted 10-fold with ChIP dilution buffer and incubated with the appropriate antibody (5 μg) at 4° for 16 h. One-fiftieth of the volume of the total extract was used for PCR amplification as the input control. Immune complexes were precipitated, washed and eluted, and DNA-protein crosslinks were reversed by heating at 65° for 4 h. The DNA fragments were purified and dissolved in 40 μl of TE. Recovered DNA was submitted for PCR amplification. The primer sequences for Rap2B are Rap2B -Ch015’-CCGAGACCAATGAGTGCAGAG-3’ and Rap2B-Ch025’-CATGCTCAGTCGC

AGAGCAG-3’. For p21, the primer sequences are p21-Ch015’-GTGGCTCTGATTGGCTT

TCT-3’ and p21-Ch025’-CTGAAAACAGGCAGCCCAAGG-3’.The PCR products generated based on the ChIP template were separated on 1% agarose gel and visualized under UV light after staining with ethidium bromide.

### Luciferase reporter assay

Luciferase reporter assay was performed using Dual-Luciferase Reporter Assay System (Promega, Madison, WI) according to the manufacturer’s instructions. Rap2B-p(1-6) promoter were subcloned into pGl3 luciferase vector (for the primer’s sequences see Supplementary Table 1). The resulting constructs were confirmed by sequencing. In brief, 100 ng pGl3-Rap2B (promoter) was co-transfected into H1299 cells with or without 10 ng pcDNA3.1-p53. Fold induction was calculated to evaluate the relative activities of the Rap2B promoter and expressed as the signal with p53 over the signal without p53. The experiments were repeated at least three times to calculate the average of fold induction.

### Measurement of intracellular IP3

The intracellular IP3 concentration in U2OS and HCT116 cells was measured by using Human inositol 1, 4, 5,-trisphosphate (IP3) ELISA Kit (Cusabio, China). After transfection, the cells were diluted to 1×100/ml with PBS, and then lysed to release intracellular components by repeated freezing and thawing. The supernatant was carefully collected after centrifuging for 20 min at 2,000-3,000 rpm. According to the manufacturer’s protocol, absorbance O.D. of each well was determined by using a microplate reader set to 450 nm. By using the professional soft “Curve Expert 1.3” to make a standard curve, the IP_3_ concentrations were calculated.

### Measurement of intracellular calcium

The intracellular calcium concentration in U2OS and HCT116 cells was examined by the Ca^2+^-sensitive fluorescent dye Fulo 3-AM (Dojindo Laboratories, Japan). After transfection and specific treatments, cells were washed with HBSS three times and incubated in 1000μl HBSS containing 5μl Fluo 3-AM for 1 h at 37°C in the dark. Subsequently, cells were washed with HBSS for three times and analyzed by using a FACSCanto flow cytometer (BD Biosciences, San Jose, CA).

### Western blot

After specific treatments, cells were incubated in lysis buffer (Roche Molecular Biochemicals, Mannheim, Germany) for 20 min on ice. After insoluble debris was pelleted by centrifugation at 14,000g for 15 min at 4°, the supernatants were collected and determined for protein content using the bicinchoninic acid (BCA) kit (Pierce, USA) according to the manufacturer’s instructions. Proteins (80μg) were resolved under denaturing conditions by SDS-PAGE (10%) and transferred onto nitrocellulose membranes. After blocking for 2 h in phosphate-buffered saline with 0.1% Tween-20 (PBST) and 3% bovine serum albumin (BSA), membranes were incubated overnight at 4° with the appropriate primary antibody in PBST containing 3% BSA. Membranes were then washed and incubated with secondary antibodies conjugated with IRDye 680 or IRDye 800 (Sigma, 1:10 000) in PBST for 2 h. The corresponding bands were imaged with Odyssey infrared imaging system (Li-COR Lincoln, NE).

### Antibodies

The following antibodies were purchased commercially: mouse anti-Mdm2 2A10 (University of North Carolina Tissue Culture and Molecular Biology Support Facility), mouse anti-actin (Neomarkers), mouse anti-p53 DO.1 (Neomarkers), rabbit anti-Rap2B (Abcam), and goat anti-p21 (C-19) (Santa Cruz Biotechnology), rabbit anti-LC3 (Novus Biologicals).

### Statistical analysis

Data are expressed as the means ± SD. The Dunnett’s t test was used to assess differences within treatment groups. All experiments were carried out at least three times. Differences were considered significant when *P* < 0.05.

## SUPPLEMENTARY MATERIALS FIGURES AND TABLE


