# Supplementary material for: p53 upregulates PLCε-IP3-Ca2+ pathway and inhibits autophagy through its target gene Rap2B
Source: Oncotarget. 2017 May 23;8(39):64657–69. doi: 10.18632/oncotarget.18112 (PMC5630284; doi:10.18632/oncotarget.18112)
Supplement: Supplementary file 1 [file oncotarget-08-64657-s001.pdf]

p53 upregulates PLCε-IP3-Ca^{2+} pathway and inhibits autophagy through its target gene Rap2B

Supplementary Materials

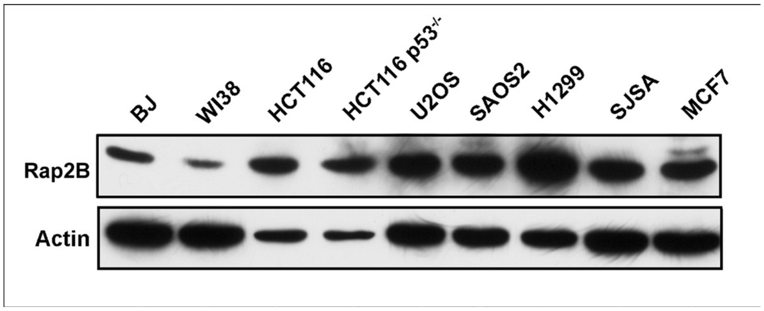

Supplementary Figure S1: Rap2B is upregulated in the tumor cell lines. Western blot analysis was performed to detect the expression of Rap2B in a couple of cell lines.

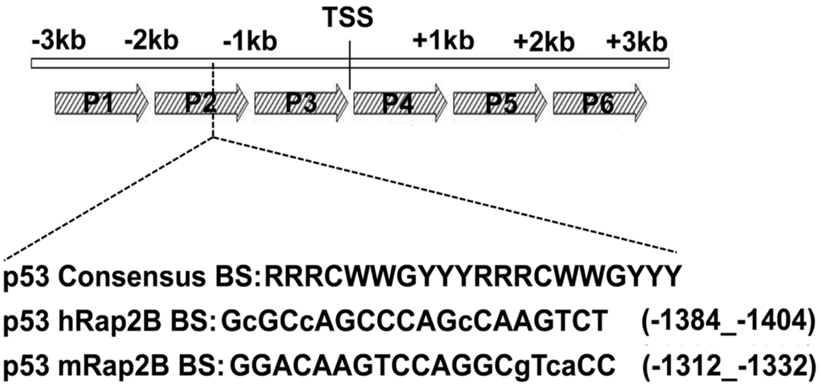

Supplementary Figure S2: A single potential binding site was identified inhuman and mouse Rap2B promoter regions. The putative p53 binding sites in the human and mouse *Rap2B* promoter regions (designated p2, corresponding to -2,000 -1,000 bp to TSS) are shown, together with the consensus p53 binding site.

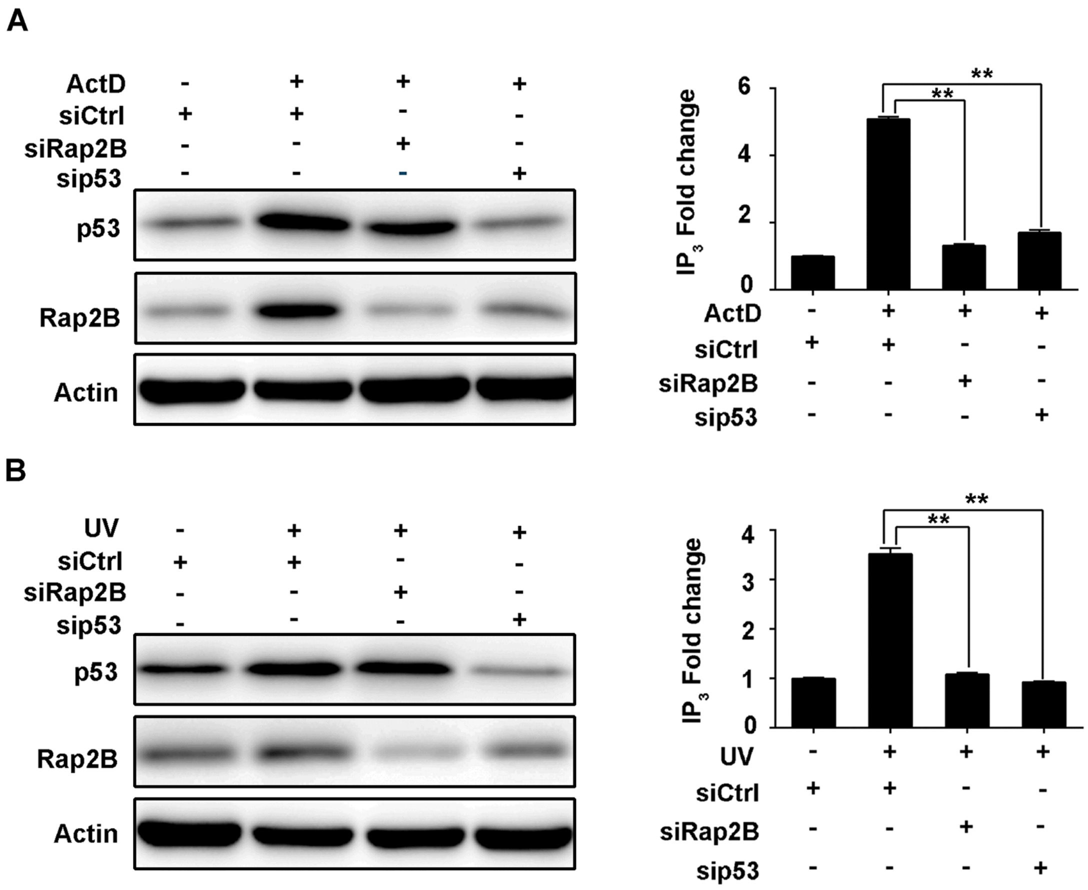

Supplementary Figure S3: p53 can increase the intracellular IP3 level through Rap2B in HCT116 cells. (A, B) After HCT116 ($p53^{+/+}$, $p53^{-/-}$) cells were transfected, cells were either treated or untreated with 5 nM ActD or 40J UV for 4 h, the protein levels of Rap2B and p53 and the intracelluar IP3 levels were measured by Western blot analysis and an IP3 ELISA Kit. All experiments were carried out in triplicate. Data are presented as mean ± SD (n = 3). $^{**}P < 0.01$ in comparison with respective control group.

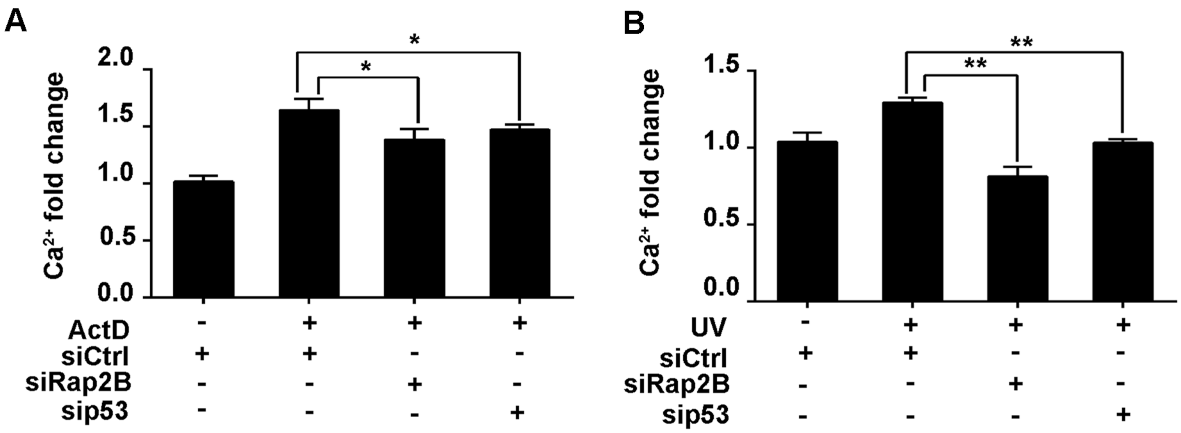

Supplementary Figure S4: p53 can increase the intracellular Ca^{2+} level through Rap2B in HCT116 cells. (A, B) After HCT116 ($p53^{+/+}$, $p53^{-/-}$) cells were transfected, cells were either treated or untreated with 5 nM ActD or 40J UV for 4 h, the intracellular Ca^{2+} levels were measured by flow cytometry. All experiments were carried out in triplicate. Data are presented as mean ± SD (n = 3). $^{*}P < 0.05$, $^{**}P < 0.01$in comparison with respective control group.

Supplementary Table 1

Primer List

pGl3-Rap2B (promoter) reporter constructs

Rap2B p1F	5’-CCGAGACCAATGAGTGCAGAG-3’
Rap2B p1R	5’-CATGCTCAGTCGCAGAGCAG -3’
Rap2B p2F	5’-CTGCTCTGCGACTGAGCATG-3’
Rap2B p2R	5’-CAGGAGGTACTCCAAGTCTGAG-3’
Rap2B p3F	5’-GCCTTAGATCTGGGAAAGTAACC-3’
Rap2B p3R	5’-GGTTGAGGCAGTGTCTACAATC-3’
Rap2B p4F	5’-CCCTCAGAAGGTCCCAGAAC-3’
Rap2B p4R	5’-GCCTTTCTTCCCCACCCAAG-3’
Rap2B p5F	5’- CGG CAC CCA CAG GAC TTC-3’
Rap2B p5R	5’- CTC TGC GAG AAG CAC GTG G-3’
Rap2B p6F	5’- CGG CCT GCA TGC TTC CTA C-3’
Rap2B p6R	5’- GAG CAT GCC CAG TGC GTT C-3’
